# Prevalence and risk factors associated with sexually transmitted infections (STIs) among women of reproductive age in Swaziland

**DOI:** 10.1186/s13027-017-0140-y

**Published:** 2017-05-25

**Authors:** Themba G. Ginindza, Cristina D. Stefan, Joyce M. Tsoka-Gwegweni, Xolisile Dlamini, Pauline E. Jolly, Elisabete Weiderpass, Nathalie Broutet, Benn Sartorius

**Affiliations:** 1 Discipline of Public Health, School of Nursing and Public Health, University of KwaZulu-Natal, 2nd Floor George Campbell Building, Mazisi Kunene Road, 4041 Durban, South Africa; 2 Walter Sisulu University, Umtata, South Africa; 3 Epidemiology Unit, Ministry of Health and Social Welfare, Mbabane, Swaziland; 4 Department of Epidemiology, University of Alabama, Birmingham, USA; 5 Department of Medical Epidemiology and Biostatistics, Karolinska Institutet, Stockholm, Sweden; 6 Department of Research, Cancer Registry of Norway, Institute of Population-Based Cancer Research, Oslo, Norway; 7 Department of Community Medicine, Faculty of Health Sciences, University of Tromsø, The Arctic University of Norway, Tromsø, Norway; 8 Genetic Epidemiology Group, Folkhälsan Research Center, Helsinki, Finland; 9 World Health Organization; Department of Reproductive Health and Research, Geneva, Switzerland

**Keywords:** Sexually transmitted infections, HIV, HPV, Risk factors, Mbabane, Swaziland, Epidemiology, Women, Cross sectional study, Africa

## Abstract

**Background:**

Sexually transmitted infections (STIs) remain an important public health problem with approximately half a billion new cases annually among persons aged 15–49 years. Epidemiological data on STIs among women of reproductive age in Swaziland are limited. The availability of epidemiological data on STIs and associated risk factors in this population is essential for the development of successful prevention, diagnosis and management strategies in the country. The study aimed to determine the prevalence and risk factors associated with STIs.

**Methods:**

A total of 655 women aged 15–49 years were systematically enrolled from five health facilities using a cross-sectional study design. Cervical specimen were tested using GeneXpert CT/NG Assays for *Chlamydia trachomatis (CT)* and *Neisseria gonorrhoeae (NG)*, GeneXpertTV Assay for *Trichomonas vaginalis (TV)*, and GeneXpert HPV Assays for hr-HPV. Blood samples were tested using Alere Determine HIV-1/2Ag/Ab Combo and Trinity Biotech Uni-Gold Recombigen HIV test for confirmation for HIV, and Rapid Plasma Reagin and TPHA test for confirmation for *Treponema pallidum* (syphilis). Genital warts were assessed prior to specimen collection. Survey weighted analyses were done to estimate the population burden of STIs.

**Results:**

The four most common curable STIs: CT, NG, TV, *Treponema pallidum* (syphilis), as well as genital warts were considered in this study. The overall weighted prevalence of any of these five STIs was 19.4% (95% CI: 14.9–24.8), corresponding to 72 990 women with STIs in Swaziland. The estimated prevalences were 7.0% (95% CI: 4.1–11.2) for CT, 6.0% (95% CI: 3.8–8.8) for NG, 8.4% (95% CI: 5.4–12.8) for TV, 1.4% (95% CI: 1.1–10.2) for syphilis and 2.0% (95% CI: 1.0–11.4) for genital warts. The overall weighted HIV prevalence was 42.7% (95%CI: 35.7–46.2). Among hr-HPV positive women, 18.8% (95% CI: 13.1–26.3) had one STI, while 6.3% (95% CI: 3.3–11.7) had multiple STIs. Risk factors associated with STIs were being employed (OR = 2.2, 95% CI: 1.0–4.7), self-employed (OR = 2.8, 95% CI: 1.5–5.5) and being hr-HPV positive (OR = 2.0, 95% CI: 1.3–3.1). Age (0.9, 95% CI: 0.8–0.9), being married (OR = 0.4, 95% CI: 0.3–0.7) and not using condoms with regular partners (OR = 0.5, 95% CI: 0.3–0.9) were inversely associated with STIs.

**Conclusion:**

STIs are highly prevalent among women of reproductive age in Swaziland. Thus, a comprehensive STIs screening, surveillance and treatment programme would be justified and could potentially lower the burden of STIs in the country.

## Background

Globally, sexually transmitted infections (STIs) remain a significant public health problem mainly in low-income countries [1, 2]. Currently, approximately half a billion new cases occur worldwide each year [3], and more than one million STIs are acquired per day [4]. Of the estimated total of 357 million incident cases of the curable STIs world-wide, 131 million are from *Chlamydia trachomatis* (CT), 78 million are from *Neisseria gonorrhea* (NG), 5.6 million are from syphilis and 143 million are from *Trichomonas vaginalis* (TV) cases [3, 4]. The World Health Organization (WHO) further estimated that 8.3, 21.1 and 59.7 million new cases of CT, NG and TV infections respectively, occur in sub-Saharan Africa (SSA), with the majority of new STIs occurring among the population aged 15 to 49 years [5].

Depending on which STI and the population, in low-income countries largely in SSA, NG, CT, TV, Human papillomavirus (HPV), herpes, and syphilis increase the risk of HIV acquisition and transmission between two to eight fold [4, 6–9]. Furthermore, the inflammation resulting from viral and non-viral STIs increases viral shedding of HIV-1 in the genital tract [10–12] and also increases the risk of HIV-1 transmission to the sex partners [13, 14]. It is estimated that the probability of HIV transmission per sexual contact is 6% if either partner has another STI other than HIV [15] as compared to 0.2% in the absence of STIs [16]. The impact of co-infection of HPV with HIV in the SSA has increased the burden of cervical diseases (such as cervical lesions and cancer), since HIV infected women have a higher prevalence of hr-HPV [17]. Furthermore, STIs such as NG and CT are the major causes of pelvic inflammatory disease (PID) and infertility in women [4]. In low-income countries, impairments associated with STIs are a major cause of mother and child mortality and morbidity in adolescence and also during pregnancy [18, 19].

Factors affecting the spread of STIs, including HIV, have been documented in many epidemiological studies across different populations [20–24]. STIs and HIV share the same behavioral, socioeconomic and demographic risk factors [20, 25], including age at first sexual intercourse, inconsistent condom use, having multiple sexual partners, female sex, being single and the partner’s sexual behaviour, location and culture. Furthermore, HIV infection increases the of other STIs [20, 24–28].

There is limited information on the prevalence and risk factors associated with STIs at population level in Swaziland, the country which has the highest prevalence of HIV infection worldwide. The availability of epidemiological data on STIs and associated risk factors in this population is essential for the development of successful prevention, diagnosis and management strategies in the country [29]. This study was therefore, conducted to determine the prevalence and risk factors associated with STIs among women of reproductive age in Swaziland.

## Methods

### Study setting and population

The study participants were women aged 15–49 years attending five healthcare facilities for routine healthcare and related services such as family planning, vaccination etc., from June to July 2015. This cross-sectional study included all women of reproductive age with a history of or who are currently sexually active and who provided written informed consent. The health care facilities (Mbabane Government hospital, Realign Fitkin Memorial (RFM) hospital, Hlatsikhulu hospital, Sithobela hospital and Siteki Public Health Unit) were located within the four political regions of Swaziland as shown in Table 1. The selection of the sites was based on the criteria of having fully functioning cervical cancer screening services, such as visual inspection with acetic acid (VIA) and cryotherapy. The purpose of including all fully functioning cervical cancer screening units was to achieve the goal of the ‘see and treat” approach. All health facilities were using the “see and treat” approach as at the time of the study. VIA was performed, and all those who were VIA-positive were treated with cryotherapy and followed for 12 months. However, these results are not reported in this article.

**Table 1:** Study sites per region and number of participants per site

Regions	Study site Code	Sites	Participants per-site
Hhohho	H01	Mbabane government hospital	182
Manzini	H02	RFM hospital	196
Lubombo	H03	Siteki Public Health Unit (PHU)	69
	H04	Sithobela Hospital	69
Shiselweni	H05	Hlathikhulu Hospital	139
Total		5	655

### Sample size

The sample size determination was based on the main research project aim, namely of establishing the burden of HPV infection and HPV-related conditions among women of reproductive age in Swaziland, where the value of the parameter(s) were not known in Swaziland. The prevalence of STIs investigated in the study was a secondary objective. Hence, the use of 50%, which assumes maximum variability and the largest possible sample size, given the predefined precision of ±5% and 95% confidence (5% type I error). Based on this a, sample size of 384 subjects was required to be used. The sample was further increased by a margin of 10% to account for potential non-response and multiplied by a design effect (D) of 1.5. The final calculated sample size of the study was 650 women. However, a total of 655 women participated to the study.

### Sampling strategy

The recruitment of participants was done in two sampling stages. Firstly, women were stratified by age (seven age-groups) and then sampled in each site using systematic random sampling (every third woman of each age), using the lottery method. The participants were selected from each site until the calculated sample size was achieved per site.

### Data collection

Prior to questionnaire administration, willing participants were given all the necessary information about the study, their potential contribution, and their risks and benefits before they signed the informed consent form. Furthermore, all necessary information was included in the study information sheet and informed consent documents. A structured standardized questionnaire was administered by trained nurses to obtain detailed data on socio-demographic characteristics, and sexual, reproductive and gynaecologic histories. Thereafter, the nurse midwife inspected the perineal, vulvar, vaginal and cervical regions of each woman for evidence of genital warts, ulcers, discharge, inflammation or tenderness, and recorded all abnormalities according to the study protocol. Lastly, the specimen collection for each of the tests was performed (see details below). All participants found to be exhibiting genital warts were treated with the WHO-recommended 0.5% podophyllin tincture. Additionally, all participants presenting with STIs on syndromic diagnosis or/and who tested positive for STIs, were treatment as per WHO STIs management guidelines [4], which have been adopted by the Ministry of Health. Thereafter, the participants were invited to return in 1 week for review purposes as recommended by the guidelines. Participants were also requested to communicate with their sexual partners their need to visit the clinic or seek treatment, using the provided tracing slip with a suspected STI code of treatment.

Data were entered using EpiData 3.02 for Windows (The Epi Data Association Odense, Denmark). Each participant was assigned a unique study identity number that was used to link the questionnaire and the biological specimens. Personal information was blinded from the researcher and was kept on site for feedback of the results to the women, and validation of data.

### Biological specimen collection and testing

After visual inspection of the vulva, a non-lubricated sterile disposable speculum was inserted and cervical cells were collected using the Xpert CT/NG/TV Endocervical swab (CT/NG/TV SWAB-50) (Cepheid, Sunnyvale, CA, 2014). After HIV pre-counselling, two 4 mL samples of blood (for HIV and syphilis testing) were collected in a vacutainer tube from participants consenting to HIV testing. All specimens were collected and transported daily to the National Referral Laboratory (NRL).

### Specimen testing

All specimens testing were tested at the NRL, Mbabane, Swaziland. Since this work is part of the HPV/Cervical cancer study, only overall weighted HPV and HIV prevalence results are presented in this paper, for a purpose of assessing the association between the curable STIs and HPV and HIV.

### CT/NG and TV testing

Cepheid Xpert CT/NG assay was used to detect *Chlamydia trachomatis* (CT) and *Neisseria gonorrhea* (NG) infection, and the Cepheid Xpert TV Assay was used to detect *Trichomonas vaginalis* (TV) (Cepheid, Sunnyvale, CA, 2014) [30, 31]. All tests were performed according to the manufacturers’ instructions. These are real-time polymerase chain reaction (PCR)-based assays for the simultaneous detection of CT/NG and TV from endocervical specimens.

### *Treponema pallidum* (syphilis)

*Treponema pallidum (*Syphilis) testing was routinely conducted on all participants who consented to HIV testing using a commercially available standard Rapid Plasma Reagin (RPR) test (Atlas Medical, Cambridge, UK) [32], following the manufacturer’s instructions. All reactive specimens were confirmed by *Treponema pallidum* Hemagglutination Assay (TPHA) (Omega Diagnostic, Scotland, UK) [33].

### HPV testing

The HPV-DNA testing was done using the GeneXpert HPV assay (Xpert HPV Assay) (Cepheid, Sunnyvale, CA, 2014) [34] according to the manufacture’s protocol. The Xpert HPV test gives results from six separate channels: (i) sample adequacy control (SAC), (ii) P1-HPV16, (iii) P2-HPV18/45, (iv) P3-HPV 31/33/35/52/58, (v) P4-HPV51/59 and (vi) P5-HPV39/68/56/66. An individual specimen can be positive for more than one probe.

### HIV testing

The Alere Determine HIV-1/2 Ag/Ab Combo test was used to detect both HIV-1/2 antibodies and free HIV-1 p24 antigen [35]. Reactive specimens were confirmed by Trinity Biotech Uni-Gold Recombigen HIV Test [36]. All participants who tested HIV positive were post-counselled and referred to health units offering the necessary HIV treatment, care and support services.

### Data and statistical analysis

Data were processed and analysed using Stata 13.0SE (Stata corp. College station, Texas, USA). Data were checked for possible errors and missing values prior to analysis. Age and region-weighted analyses were done to estimate the overall STIs’ prevalence and coinfection with HIV and hr-HPV.

Survey weighted analysis was done to adjust the sample characteristic to match the target population (15–49) that they were selected to represent. It is applied to bring the proportion of women in the sample in alignment with the portion of women in the target population. Therefore, all results are reported as weighted. Survey weighted prevalence and 95% confidence intervals (CI) were calculated. In addition, assuming that our study subjects were representative of the female population of the same age strata, we used survey weights to extrapolate sample proportions to population totals (age-region weighted) to estimate burden counts, based on the 2007–2030 population projections aligned to the estimated 2014 population of 377 169 women aged 15–49 years [37]. Differences in prevalence by categorical variables such as site, age, HIV and hr-HPV were assessed using the survey weighted chi-square (*χ* ^2^) test. Odds ratios (unadjusted and adjusted) and 95% CIs for potential risk factors associated with STIs were estimated using survey weighted logistic regression models. Variables that were significant at a cut-off of 0.2 in the bivariate regression analyses were selected for inclusion into the final multivariable model. An adjusted *p*-value of <0.05 was deemed statistically significant.

## Results

### Characteristics of the study population

A total of 655 women were enrolled in the study within the period of June - July 2015. All the participants had sufficient specimens for the four non-viral STIs testing and only 11 had sufficient specimen for hr-HPV. Table 2 summarizes the key characteristics of the study population. The mean ages (± standard deviation [SD]) for enrolled women was 32.2 (±8.7) years. Their mean age at menarche was 14.4 (±1.7) years, at first intercourse was 17.9 (±2.9) years, and at first pregnancy was 19.4 (±3.9) years. Of the 655 participants, 571 (88.7%) had been pregnant previously, 542 (84.2%) had a history of contraceptive use, 272 (42.6%) had a history of STIs, 116 (18.0%) had STIs that had been treated in the past 12 months, 345 (53.6%) were married or cohabiting, 227 (35.3%) had not completed secondary/high school education, 340 (53.0%) were unemployed and 513 (79.7%) reported one lifetime sexual partner.

**Table 2:** Socio-demographic characteristics of the study population (*N* = 655)

Demographics	n (%)
Age: n (%)	
Mean ± SD (range)	32 .2 ± 8.7
15–19	39 (6.1)
20–24	111 (17.2)
25–29	131 (20.3)
30–34	116 (18.0)
35–39	103 (16.0)
40–44	80 (12.4)
45–49	75 (11.6)
Marital status: n (%)	
Single	266 (41.3)
Cohabiting	38 (5.9)
Married	307 (47.7)
Divorced/separated	22 (3.4)
Widow	11 (1.7)
Education: n (%)	
Never been to school	24 (3.7)
Primary	130 (20.2)
Secondary/High	373 (57.9)
Tertiary	117 (18.2)
Occupation: n (%)	
Unemployed	340 (53.0)
Employed	255 (39.8)
Self-employed	46(7.2)
Ever pregnant : n (%)	
Yes	571 (88.7)
No	64 (9.9)
Missing	9 (1.4)
Age at first pregnancy: Mean age (SD)	19.4 (3.9)
No. pregnancies: n (%)	
0	6 (1.0)
1	139 (24.3)
2	127 (22.2)
3+	297(46.1)
Age Menarche: Mean (SD)	14.39 (1.7)
Age at first intercourse: Mean age (SD)	17.90 (2.9)
Number of Sexual life partner: n (%)	
0	33 (5.1)
1	513 (79.7)
2	61 (9.5)
3+	43 (6.7)
Currently using contraceptives: n (%)	
Yes	542 (84.2)
No	95 (14.8)
Missing	7 (1.1)
Ever had STI: n (%)	
Yes	272 (42.6)
No	337 (52.7)
Don’t remember	30 (4.7)
STIs treated in the past 12 months: n (%)	
Yes	116 (18.0)
No	520 (80.9)
Don’t know	7 (1.1)

### Prevalence of STIs

Table 3 shows the weighted prevalence of selected STIs in the samples of women aged 15–49 years. The overall STIs’ weighted prevalence (excluding HIV and HPV) was 19.4%, (95% CI: 14.9–24.8) and individual prevalence for *Trichomonas vaginalis* (TV), *Neisseria gonorrhoeae* (NG), *Chlamydia trachomatis* (CT), *Treponema pallidum* (syphilis) and genital warts (GW) was 8.46.0, 7.0, 1.4 and 2.0%, respectively. The overall weighted hr-HPV prevalence and HIV prevalence was 46.2% (95% CI: 42.8–49.5), and 42.7% (95% CI: 35.7–46.2) respectively, (Table 3) (detailed data shown in a previously published article [38]). About 4.0% (95% CI: 2.3–6.0) had two or more STIs (multiple infections) and 6.0% (95% CI: 4.3–8.5) had triple infection (at least one STI, HIV and hr-HPV infection) (Table 3). Among women with hr-HPV, 18.9% (95% CI: 13.1–26.3) had a single STI and 6.3% (95% CI: 3.3–11.7) had multiple STIs. Among HIV positive women, 17.7% (95% CI: 11.5–26.9) had one STI, while 4.2% (95% CI: 1.5–11.3) had multiple STIs (Table 4). About 6.3% (95% CI: 4.4–8.9) women had STIs, HPV and HIV combined coinfections.

**Table 3:** The prevalence of sexually transmitted infections (STIs) among women aged 15–49 in Swaziland (*n* = 655)

STIs	Positive (n)	Crude Prevalence (%)	Survey weighted prevalence % (95% CI)	Population burden^a^	95% CI
Overall	114	17.4	19.4 (14.9–24.8)	72990	51111–94871
TV	51	4.7	8.4 (5.4–12.8)	30132	16633–43631
NG	35	5.2	6.0 (3.8–9.4)	22055	11986–32124
CT	38	5.8	7.0 (4.1–11.2)	25024	11251–38797
*Treponema pallidum* (syphilis)	9	1.2	1.4 (1.1–10.2)	5072	1822–8322
Genital warts	6	1.0	2.0 (1.0–11.4)	6111	470–12692
HIV	276	42.1	42.7 (35.7–46.2)	153276	127336–179216
Hr-HPV (*n* = 644)	273	42.1	46.2 (42.8–49.5)	174046	153294–194797
Multiple STIs^b^	20	3.1	4.0 (2.3–6.0)	13995	7092–20898
^c^Triple STIs (*n* = 644)^d^	38	5.9	6.3 (4.4–8.9)	23310	14193–32427

^a^Population burden estimates extrapolated (using sample statistics) based on the 2007–2030 population projections aligned to the 2014 population estimates

^b^Multiple STIs – having two or more of the screened 5 STIs

^c^Triple infection: at least one other STI including HIV and hr-HPV infection

^d^A total of 11 women had insufficient specimen for hr-HPV testing therefore for the analysis for Triple STIs done on *N* = 644

**Table 4:** The weighted prevalence and estimated population burden of other STIs and hr-HPV/HIV co-infection among women of reproductive age in Swaziland

Characteristic	hr-HPV status(*N* = 644)^a^			HIV status(*N* = 654)^b^		
	Negative (%, 95% CI)	Positive (%, 95% CI)	Total	Negative (%, 95% CI)	positive (%, 95% CI)	Total
STIs status						
Negative	86.3 (79.4–91.1)	74.8 (67.3–81.1)	81.1(75.6–85.4)	82.2 (72.8–92.8)	78.1 (66.5–90.0)	80.6 (72.1–89.7)
Single	12.1 (7.4–19.1)	18.9 (13.1–26.3)	15.2 (11.8–19.4)	14.4 (9.7–22.6)	17.7 (11.5–26.9)	15.7 (11.2–22.6)
Multiple	1.6 (0.7–3.4)	6.3 (3.3–11.7)	3.7 (2.3–6.1)	3.4 (2.0–5.7)	4.2 (1.5–11.3)	3.7 (2.3–6.0)
Total	100	100		100	100	100
	*p*-value = 0.025			*p*-value = 0.370		
Population Burden						
STIs status	Negative (%, 95% CI)	Positive n(95% CI)	Total	Negative (%, 95% CI)	Positive n(95% CI)	Total
Negative	171725 (145875–197575)	127553 (110677–144428)	299278 (265003–333552)	183121 (148333–217908	119746 (90801–148690)	302867 (39415–348608)
Single	24073 (11482–36665	32158 (20200–44115)	56231 (39657–72806)	31832 (16847–46490)	27163 (14941–39386)	58996 (35732–82259)
Multiple	3197 (671–5723)	10798 (4032–17564)	13995 (7092–20898)	7628 (3919–11336)	6367 (−159–12893)	13995 (7092–20898)
Total	198996 (169168–228822)	170508 (154052–186965)	369504	222581 (190953–254208)	153276 (127336–179216)	357857
	*p*-value = 0.025			*p*-value = 0.370		

other STIs (CT, NG, TV, syphilis, Genital warts)

Single STI: one of the 5 STIs screened or observed

Multiple STIs: two or more of the 5 STIs screened or observed

^a^ *N* = 644 : A total of 11 women had insufficient specimen for hr-HPV testing

^b^ *N* = 654 : one with unknown/missing HIV status

### Population burden estimates

Tables 3 and 4 show the population burden estimates. Population burden estimates to extrapolate absolute burden counts of the different STIs were made based on the 2007–2030 population projections aligned to the 2014 population estimates of women aged 15–49 years. The overall population burden was estimated at 72 990 (95% CI: 51 111–94 871) as depicted on Table 3. When stratified by each STI, 30 132 (95% CI: 16 633–43 631) were estimated to have TV, 22 055 (95% CI: 11 986–32 124) have NG, 25 024 (95% CI: 11 251–38 797) have CT, 5 072 (95% CI: 1 822–8 322) have *Treponema pallidum* (syphilis), and 6111 (95% CI: 470–12692) have GW. Multiple STIs estimates were 13995 (95% CI: 7 092–20 898) and triple STIs were 23 310 (95% CI: 14 193–32 427). Among HIV positive women, the population burden counts for single STIs were 32 158 (95% CI: 20 200–44 115) and for multiple infection 10 798 (95% CI: 4 032–17 564). Among hr-HPV positive women the population burden counts for single and multiple infection were 27 163 (95% CI: 14 941–39 386) and 6 367 (95% CI: 159–12 893), respectively) (Table 4).

### Prevalence of STIs by selected socio-demographic characteristics of the women

Table 5 shows the prevalence of STIs by selected socio-demographic characteristics of the women, and the risk factors associated with STIs (univariable and multivariable logistic regressions). The prevalence of STIs decreased with increasing age, with the highest burden among the 15–19 and 24–29 age groups (29.2 and 27.0% respectively) (Table 5). There was no statistically significant difference between age-group observed for all or individual STIs, although the prevalence of CT was slightly higher among women aged 15–19 years (12.1%, 95% CI: 3.4–35.5, *p* = 0.32), NG was higher among 20–24 years old (12.1%, 95% CI: 5.4–25.1, *p* = 0.41) while TV was higher among 25–29 years old (13.9%, 95% CI: 10.6–18.1, *p* = 0.15) as compared to all ages (data not shown).

**Table 5:** Risk factors associated with STIs among women aged 15–49 years in Swaziland (*N* = 655)

Risk factors	STIs (Weighted Prevalence)		Unadjusted (Univariate)		Adjusted (Multivariable)	
	Number + ve/Total	(%, 95% CI)	OR (95% CI)	*P*-value	OR^a^ (95% CI)	*P*-value
Overall	114/655	19.3 (14.9–24.8)				
Age						
Mean ± SD (range)	114/655	27 .1 (25.9–28.2)	**0.9 (0.8–0.9)**	**0.001**	**0.9 (0.8–0.9)**	**<0.001**
15–19	13/45	29.2 (14.3–59.4)	**1 (ref)**			
20–24	26/110	22.7 (13.1–37.4)	0.7 (0.2–2.1)	0.520	….	….
24–29	34/127	27.0 (19.0–38.1)	0.9 (0.4–2.1)	0.797	…..	….
30–34	20/143	13.0 (6.0–3161)	0.4 (0.1–1.1)	0.081	….	….
35–39	14/94	14.5 (7.7–28.8)	0.4 (0.1–1.2)	0.110	....	….
40–44	6/78	6.9 (1.5–29.9)	0.2 (0.03–1.1)	0.067	….	….
45–49	1/58	1.0 (0.1–8.7)	0.03 (0–0.3)	0.004	….	….
Marital status						
Single	66/271	26.4 (18.5–37.4)	**1 (ref)**			
Living with partner/Cohabiting	9/38	24.2 (9.8–62.3)	0.89 (0.3–2.5)	0.819		
Married	36/313	11.2 (6.7–18.9)	**0.35 (0.2–0.6)**	**0.001**	**0.4 (0.3–0.7)**	**0.001**
Divorced/separated	3/22	15.1 (4.9–38.3)	0.5 (0.13–1.9)	0.289	….	….
widow	0/11	0	**---**	**---**	….	….
Education:						
Never been to school	2/24	9.4 (19.0–36.0	**1 (ref)**			
Primary	23/133	21.4 (10.5–47.8)	2.6 (0.4–16.9)	0.299	….	....
Secondary/High	78/380	22.2 (15.4–31.3)	2.7 (0.5–15.4)	0.243	….	…
Tertiary	11/118	7.3 (3.5–16.9)	0.8 (0.1–5.4)	0.777	….	….
Occupation:						
Unemployed	67/346	21.6 (14.2–32.9)	**1 (ref)**		1 (ref)	
Employed	39/258	14.7 (9.8–22.0)	0.63 (0.34–1.16)	0.129	**2.2 (1.0–4.7)**	**0.045**
Self-employed	8/48	21.8 (8.0–47.4)	1.01 (0.32–3.19)	0.982	**2.8(1.5–5.5)**	**0.002**
Pregnant before						
Yes	99/582	18.3 (13.5–24.4)	**1 (ref)**			
No	13/64	25.2 (12.6–48.9)	1.51 (0.65–3.46)	0.323	….	….
No. of pregnancies (Mean)	114/655	2.2 (1.9–2.5)	**0.8 (0.7–0.9)**	**0.008**	….	…
Age Menarche (Mean age)	114/655	13.97 (13.5–14.4)	0.9 (0.7–1.1)	0.166	….	..
Age at first sex (mean age)	114/655	17.2 (16.5–17.9)	0.96 (0.9–1.1)	0.370	…..	…..
Number of sexual partners		2.0 (0.6–3.4)	**1.3 (1. 4–1.5)**	**<0.001**	1.2 (0.9–1.5)	0.197
0	3/33	6.2 (14.2–23.6)	**1 (ref)**		….	…
1	84/522	18.4 (14.2–23.6)	3.4 (1.0–12.3)	0.056	….	….
2	16/62	25.1 (11.3–46.8)	**5.1 (1.1–21.5)**	**0.028**	….	….
3+	11/37	35.6 (15.7–53.3)	**8.4 (1.3–54.2)**	**0.027**	….	….
Condom use with regular partner						
Yes	93/457	21.6 (14.8–31.2)	1 (ref)		1 (ref)	
No	20/194	13.1 (5.7–29.1)	**0.5 (0.28–0.9)**	**0.022**	**0.5 (0.3–0.9)**	**0.034**
Ever had STI						
Yes	41/278	16.2 (10.9–23.4)	**1 (ref)**			
No	63/342	19.8 (15.2–25.3)	1.28 (0.8–2.0)	0.246	….	….
Don’t remember	7/30	31.8 (12.0–61.3)	2.42 (0.7–7.8)	0.136	….	….
STIs treated in the past 12 months						
Yes	24/118	22.6 (13.5–35.5)	**1 (ref)**			
No	89/529	18.7 (14.7–23.6	0.8 (0.5–1.4)	0.376	….	…..
Don’t know	1/7	13.9 (12.6–67.3)	0.6 (0.4–8.1)	0.654	….	….
HIV Status:						
Negative	55/373	17.7 (13.4–23.0)	**1 (ref)**			
Positive	59/281	21.9 (15.2–30.4)	1.3 (0.9–2.0)	0.204	…..	….
hr-HPV status:						
Negative	46/371	13.7 (8.9–20.6)	1 (ref)		1 (ref)	
Positive	64/273	25.2 (19.0–32.7)	**2.1 (1.3–3.6)**	**0.007**	**2.0 (1.3–3.1)**	**0.002**

The Bolded data shows the signficant results

^a^Adj. OR: Adjusted Odds Ratio for age, marital status, level of education, occupation, history of pregnancy, age at first sex, history of STIs, STIs treated in the past 12 months, HIV status

The prevalence of STIs was significantly higher among single women (26.4%) as compared to married women (11.2%) (*p* = 0.001). Women with secondary/high school education had a prevalence of 22.2% as compared to women who had never been to school (9.1%). Unemployed and self-employed women had a prevalence of 21.6, and 21.8% respectively as compared to employed women (14.7%). There was no statistically significant difference between women who reported being pregnant before as compared to women who had never been pregnant (25.2% vs 18.3%, *p* = 0.323).

Based on the univariate analysis: the mean age, being married, increasing number of pregnancies and not using condoms with ones’ regular partner were inversely associated with STIs’ risk. Significant differences in proportions regarding those married (*p* < 0.001), the number of sexual partners (*p* < 0.001) and contraceptive use by age group (*p* < 0.001) was observed. Having two and three sexual life-time partners was associated with an increased risk OR = 5.1 (95% CI: 1.2–21.5) and OR = 8.4, (95% CI: 1.3–54.2) respectively for STIs. Being hr-HPV positive was significantly associated with increased risk of STIs (OR = 2.1, 95% CI: 1.3–3.6). In the final logistic regression model (multivariate analysis), factors independently associated with increased risk of STIs were: being employed (OR = 2.2, 95% CI: 1.0–4.7), self-employed (OR = 2.8, 95% CI: 1.5–5.5) and hr-HPV positive status (OR = 2.0, 95% CI: 1.3–3.1). Being married, mean age and not using a condom one’s with regular partner were found to be inversely associated in the final model (OR = 0.4, 95% CI: 0.3–0.7, OR = 0.92, 95% CI: 0.89–0.95, and OR = 0.5, 95% CI: 0.3–0.9) respectively, as compared to not having a sexual life partner.

Individual STI association with HIV and hr-HPV was assessed. There was a significant inverse association between HIV and CT (*p* = 0.001) but there was a significant positive association between HIV and syphilis (*p* = 0.001). There was a significant positive association between hr-HPV and NG and CT (*p* = 0.020 and *p* = 0.001). A significant association with TV and hr-HPV (*p* = 0.058) was observed (results not shown).

## Discussion

The current study in Swaziland shows a high prevalence of STIs (19.4%), corresponding to 72 990 women of reproductive age with STIs. Our data suggests that TV was the most prevalent curable STI (8.4%) among those examined in this study. Furthermore, the prevalence of HIV remains significantly high (42.7%) in this population. The prevalence of the STIs decreased with increasing age however, differences by age groups were not statistically significant. A high percentage of women infected with hr-HPV had one STI (18.8%), while 6.3% had multiple STIs. Single women were at higher risk of being infected with STIs compared to married women (26.4% vs 11.2%, *p* = 0.001). A proportion of the women had triple co-infection (6.3%), interact either directly with one another or indirectly via the host’s resources or immune system. Studies have demonstrated that, as compared to infections of single pathogen species, these interactions within coinfected hosts can alter the transmission, clinical progression and control of multiple infectious diseases [39, 40]. In the final logistic regression model, being employed, self-employed and hr-HPV infection status were risk factors positively associated with STIs.

In this study, we collected primary data and biological specimens using a standardized methodology. This is the first study evaluating the prevalence of STIs among women of reproductive age not attending antenatal care in Swaziland. We were able to extrapolate our results on the prevalence of STIs as well as genital warts to the female population in the same age groups of 15–49 years-old using the 2007–2030 population projections aligned to the 2014 population estimates [37]. Another strength of our study is that STIs such as NG, TV and CT were detected using the Gene Xpert PCR (Xpert CT/NG and Xpert TV) which, is a highly sensitive laboratory method for detecting genital infection. An important limitation of the study is the lack of generalizability of the results to the general Swazi female population beyond the age groups studied (15–49 years). In addition, since the study subjects were recruited from heath care facilities, it is arguable that they may not be truly representative of the general population. This could have introduced a selection bias. However, weighting was applied when reporting the summary results for the whole study sample. Lastly, the study participants were required to recall past events, which could result in compromising the accuracy of information provided, due to recall bias. Finally, due to limited resources for our study we did not genotype or test for LR-HPVs.

The high prevalence of STIs (19.4%) in this population indicates that STIs are a serious public health problem in Swaziland. Our findings were consistent with previous studies from similar populations across the Southern African Development Community region (SADC) and other sub-Saharan regions [13, 15, 41–43]. The prevalence of each of the STIs detected among the study population was less than 10% (7.0% for CT, 6.0% for NG, 8.4% for TV and 1.4% for syphilis), but this is still high, which compared to global estimates, where the prevalence for CT among women range from 2.4 to 6.9%, NG from 0.3% to 1.2, TV from 1.9 to 7.8% and syphilis from 0.2 to 1.3 [44]. Results from the firstand second STI sentinel surveillance surveys conducted in 2003 and 2005 respectively in Swaziland, showed that the prevalence of STIs, such as CT, NG, TV and syphilis, and other reproductive tract infections, such as bacterial vaginosis and candidiasis, continue to be among the highest in the country [45, 46]. However, a decline in syphilis has been noted in recent years. According to the 11th Sentinel Surveillance report 2008, recent syphilis infection was tested using *Treponema pallidum* haemagglutination (TPHA), and the prevalence was 1.9% while 4.7% showed a history of syphilis infection by RPR results [47]. A comparison between the 2008 report and recent syphilis infection shows a decreasing trend (1.9%) compared to current study findings (1.4%). The high prevalence of the curable STIs in this population might be linked to the limitation of syndromic STI management, which does not give specific aetiological diagnosis; therefore, most infections are missed by this approach [48, 49]. Although syndromic management has proven to be cost-effective in resource limited settings, our findings demonstrate the evidence for the need of a strong national STIs’ surveillance system.

HIV remains one of the major public health challenges in Swaziland, with the highest prevalence among women of reproductive age [50, 51]. This high prevalence reported in our study was consistent with other studies. However, our results were slightly higher as compared to Bicego et al’s study on recent patterns in population–based HIV among women age 18–49 years (42.7% vs. 39%) [50]. In a country that has the highest HIV prevalence in the world, the challenge remains to assist uninfected individuals in gaining access to high quality prevention services, including early detection and treatment of curable STIs.

The observed high prevalence of STIs in the younger age groups as compared to the older age groups in our study is consistent with other studies [41, 52, 53]. The decreasing STIs prevalence with increasing age observed in our study, might be the results of the high susceptibility of younger women, lack of protective immune response development, and more risky behavior, such as an earlier age at first intercourse, a high number of sexual partners, and unprotected sex [52].

In this current study, single women and women living with partners (but not married) had the highest prevalence of STIs. Similar evidence has been found in other studies [54, 55]. In our multivariate analysis, married women were 60% less likely to be at risk of STIs as compared to single women (OR = 0.4). A similar outcome was found in others studies showing being married is a protective factor [48, 56].

As far as education is concerned, our findings have shown that women with a lower level of education are most likely to have an STI. Studies have shown that women with less education have an increased risk of STIs [13, 57, 58]. Kakaire et al. highlighted that women with lower education tend to lack formal employment and may be completely dependent on the male sexual partner and unable to negotiate safer sex. They are less likely to access STI preventative information and healthcare services [13]. According to Muula, unemployment may leave women with limited alternatives where they may resort to engaging in high risk behavior such as becoming sex workers or to engaging in transactional sex in which they provide sex in exchange for money and material resources from a partner [59]. However, our study found that employed and self-employed women were at high risk of STIs (OR: 2.2 and OR: 2.8 respectively) as compared to unemployed women. The types of employment might explain the high risk of STIs among employed and self-employed women in our study and the in which businesses they are involved. There is evidence suggesting that women working in high-risk occupation such bars, food facilities, guesthouses and similar facilities are at high risk of STI infection [48].

In this study, the risk associated with STIs among women with two or more sexual life-time partners was five to eight times higher as compared to women with no sexual partners in the univariate analysis. Our findings were consistent with other studies which reported that sexual activity with many partners increases the odds of STIs [53, 54]. However, the association was not statistically significant in our study following adjustment for all possible confounding factors (age, marital status, level of education, occupation, history of pregnancy, age at first sex, history of STIs, STIs treated in the past 12 months, and HIV status), which negatively confounded this relationship. There was a very high collinearity between the number of sexual partners and some of the confounding factors, which may have further affected the coefficients in the stepwise multivariable regression. Though evidence from studies done in South Africa [41, 42], Zambia [60], and Tanzania [48] has demonstrated that having more than one or concurrent lifetime sexual partners was among the risk factors associated with STIs.

Inconsistent condom use as a risk factor for STIs in women has been demonstrated in other studies [61, 62]. In contrast to our study, not using condoms with regular partners was inversely associated with STIs. The possible plausible explanation of our findings might be that, women who reported not using condoms with regular partners had one faithful partner or they had been treated for STIs prior to data collection. However, this will require further investigation. Moreover, condoms remain a critical part in a comprehensive and practical approach to the prevention of STIs [63].

HIV positive status was not associated with STIs in both analyses but when assessed by individual STIs, a significant inverse association between HIV and CT, and significant positive association between HIV and syphilis were observed. No statistically significant association between HIV and NG or TV was observed in the current study. However, studies have indicated that depending on the STI involved and the population, NG, CT, TV, HPV and syphilis increase the risk of HIV acquisition and transmission from two to eight times or more [4, 6, 7]. Furthermore, biological findings support the mechanisms for STIs increasing HIV acquisition and transmission through direct mucosal disruption, recruitment of HIV target cells to the genital tract, and by increased HIV load in plasma and genital secretions [10]. High levels of untreated STIs in sub-Saharan countries are linked to high HIV transmission rates and have been postulated to have contributed to the high prevalence of HIV in the region [16].

A vital clinical and public health consequence of our findings is that hr-HPV was the most significant risk factor associated with STIs infection in both univariate and multivariate analysis (unadj-OR = 2.1 and Adj-OR = 2.0). When assessing the association between hr-HPV and individual STIs, there was a significant positive association between hr-HPV and NG and CT. The strong association between HPV infection and STIs has been demonstrated in previous studies, where infection with hr-HPV types, was a risk factor for CT and NG infection [64–66]. These findings are significantly of high importance for policy and prevention programmes to include HPV infection in the existing STIs prevention programmes in the country.

The curable STIs (CT, NG, TV, and syphilis), and HIV and HPV are sexually transmitted infections, which share the same transmission route and behavioural risk factors. If the curable STIs are not treated, they can lead to a variety of different health risks and greatly increase HIV/HPV transmission risk with further chronic health implications. Collectively, our data have shown the significantly high prevalence of and association between STIs and HIV/HPV coinfection, which tends to subscribe to the new concept in which HIV and HPV infections may be bi-directional, each increasing the risk of the other [67–69] STIs increase the risk of HIV/HPV co-infection. The high hr-HPV/HIV coinfection with the curable STIs and resultant high population burden has implications for both cervical cancer and also for the United Nations (UN) sustainable development goal 3, target 3.3 (SDG 3) [70] (goal 3 aims to reduce new HIV infection through ensuring health and well-being for all, at every stage of life). The STIs are positively increasing the risk of HIV/HPV acquisition, which will make it impossible for countries like Swaziland to achieve SDG 3 target 3.3. Therefore, STIs’ control and HPV vaccination, combined with periodic HIV screening and referral to early treatment is needed to end the HIV epidemic in Swaziland.

## Conclusion

In conclusion, although STIs may be treated and are curable, they remain a major public health challenge in Swaziland where HIV infection is highly prevalent. Our study indicates that there is a need for a comprehensive STI screening, surveillance and treatment programme in the country.

## Abbreviations

CT: Chlamydia trachomatis
HIV: Human immunodeficiency virus
Hr-HPV: High-risk human papillomavirus
NG: Neisseria gonorrhoeae
NRL: National referral laboratory
RFM: Realign Fitkin Memorial Hospital
SDG: United Nations Sustainable Development Goal
STIs: Sexually transmitted infections
TPHA: Treponema pallidum Hemagglutination Assay
TV: Trichomonas vaginalis
VIA: Visual inspection with acetic acid
